# Tissue adhesive for wound closure in enhanced-recovery total hip arthroplasty: a prospective, randomized and controlled study

**DOI:** 10.1186/s12891-020-03205-5

**Published:** 2020-03-19

**Authors:** Xiangpeng Kong, Minzhi Yang, Zheng Cao, Jiying Chen, Wei Chai, Yan Wang

**Affiliations:** 1 Department of Orthopaedics, Chinese PLA General Hospital, No.28 Fuxing Road, Haidian, Beijing, China; 2 Nankai University, No.94 Weijin Road, Nankai, Tianjin, China

**Keywords:** Total hip arthroplasty, Enhanced recovery after surgery, Tissue adhesive, Wound closure, Dressing change, Prospective, randomized and controlled study

## Abstract

**Background:**

We aimed to present our experience of adopting tissue adhesive as adjunct to standard wound closure in total hip arthroplasty (THA) and evaluate its performance.

**Methods:**

From September 2019 to November 2019, we prospectively enrolled consecutive patients who underwent simultaneous bilateral THA in this randomized and controlled study. Standard wound closure was applied on one side of hip while additional tissue adhesive was applied on the other side at random. We collected and analyzed patients’ information, including age, gender, body mass index (BMI), diagnosis, postoperative length of stay (LOS), dressing changes, wound evaluation scores, wound-related cost and complications.

**Results:**

Thirty patients with simultaneous bilateral THA were enrolled in this study. During the hospital stay, the times of dressing change in hips with tissue adhesive was significantly less than that in the other hips (*p* = 0.000). However, the wound-related cost in hips with tissue adhesive was significantly higher (*p* = 0.000). According to patients’ feedback at one-month follow-up, wound evaluation of hips with tissue adhesive was significantly better than the other hips (*p* = 0.004). Seventeen patients preferred tissue adhesive and only five patients preferred standard wound closure.

**Conclusions:**

Tissue adhesive could significantly reduce wound drainage and increase patients’ satisfaction, which can be an ideal adjunct to standard wound closure in enhanced-recovery THA.

**Trial registration:**

Chinese Clinical Trial Registry; ChiCTR1900025730; Registered 6 September 2019.

## Background

At present, enhanced recovery after surgery (ERAS) has developed rapidly in the field of joint replacement [1–3]. In some institutes, the length of stay (LOS) after total hip arthroplasty (THA) has been shortened to less than 48 h and some even became daytime surgery [4, 5]. The advent of ERAS also raised the stringent requirements of surgical techniques and perioperative management [6, 7]. Wound closure is one of the most important aspects in perioperative management. However, compared with surgical techniques, there were relatively fewer studies on wound closure and care.

Prolonged wound drainage, which is a common complication after joint replacement, could result in delayed wound healing, limited postoperative activity and even periprosthetic joint infection (PJI) [8, 9]. One ideal wound closure should be designed simple and convenient.

In recent years, tissue adhesive has been introduced and adopted in orthopedic surgery [10–15]. According to the previous studies, tissue adhesive may be an ideal supplement to standard wound closure following total knee arthroplasty (TKA) [8]. It exists as liquid and can polymerize rapidly when contacting with skin tissue. The protective film produced by the tissue adhesive could quarantine with the external environment and prevent the foreign substances from invading the wound in the early postoperative period [16–19]. As we know, there was no study ever focused on the application of tissue adhesive as the supplement to subcuticular suture in THA.

Thus in this prospective study, we aimed to present our experience of adopting tissue adhesive as adjunct to standard wound closure in THA and evaluate its role and cost performance.

## Methods

### Study population and design

From September 2019 to November 2019, we prospectively enrolled consecutive patients who underwent simultaneous bilateral THA in this randomized self-control study. The study was approved by the local ethics committee.

Inclusion criteria: 1. age between 18 and 60 years old; 2. the bilateral THA with the same prosthesis through the posterolateral approach; 3. the written informed consent obtained prior to participating in this study. Exclusion criteria: 1. previous open surgery or major trauma or infection in either hip; 2. eloid, psoriasis, eczema or other skin diseases; 3. allergy to the ingredients of the tissue adhesive; 4. underlying malignant tumors; 5. regular anticoagulation therapy; 6. peripheral vascular disease; 7. active inflammatory arthropathy; 8. bilateral surgeries performed in stages.

Sample size calculation: according to the previous study and preliminary results of our pre-experiment, we set α = 0.05, β = 0.10, the mean difference of times of dressing change was 1.0. An estimated 24 patients would be needed to provide 90% power. In the end, we decided to enroll 30 patients, which allowed for 20% loss to follow up.

### Surgical procedures of wound closure

All patients underwent THA on right side firstly. The fixed surgical team performed surgeries and two fixed residents performed wound closure. Tranexamic acid (TXA) was given intravenously twice before incision and wound closure.

Standard wound closure for different layers: 1. joint capsule and external rotator muscles were reconstructed with 2–0 Ethibond non-absorbable suture W4843 (Ethicon, Somerville, NJ, USA). 2. deep fascia and superficial fascia were sutured with 2–0 absorbable knotless barbed running suture (Quill, Surgical Specialties Corporation, IL, USA) and 4–0 coated Vicryl Plus antibacterial interrupted suture (Ethicon, Somerville, NJ, USA). 3. subcuticular tissue was closed with 4–0 absorbable knotless barbed running suture (Ethicon, Somerville, NJ, USA).

**Fig. 1:**
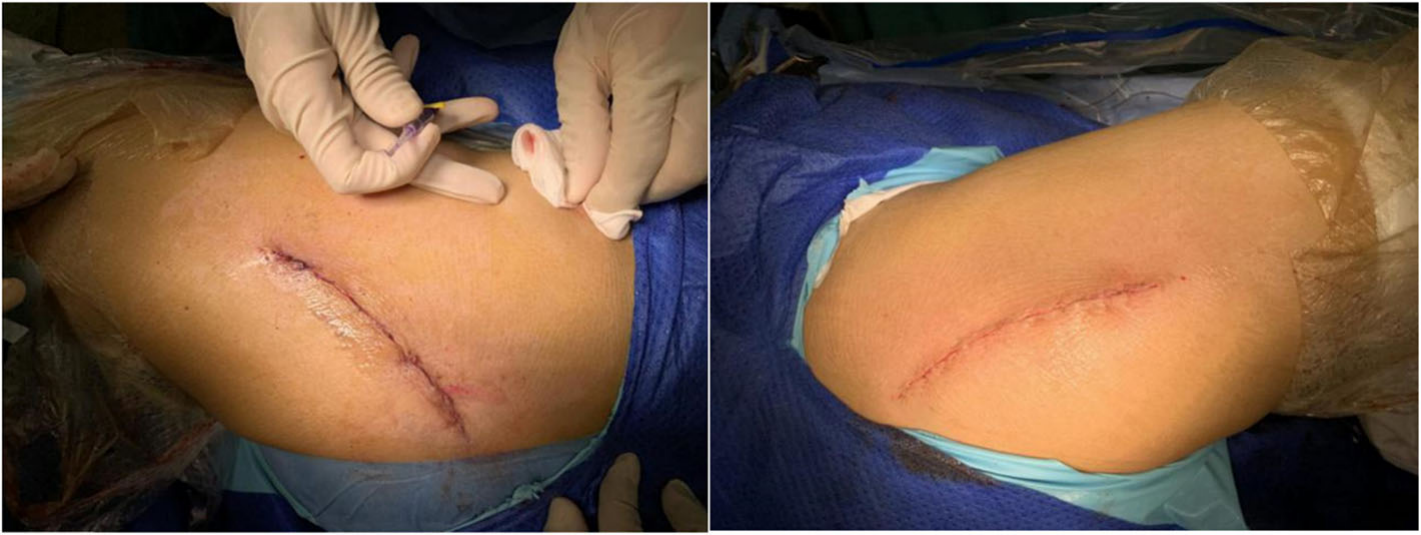
The appearances of bilateral wounds in operating room (left: tissue adhesive; right: standard wound closure)

The right hip was randomized by computer-generated method in the opaque envelopes after standard wound closure. Standard wound closure was applied on one side of hip while additional tissue adhesive was applied on the other side (Fig. 1). The residents remained blinded before the allocations.

The HISTOACRYL® tissue adhesive (B.Braun, Melsungen, German) was applied evenly on the wound and waited air-drying for 30 s. When tissue adhesive got dry, wound was covered by wound dressing. During the hospital stay, patients and caregivers were told to inform nurses of dressing change if blood or exudate soaked the dressing, which was the standard protocol of postoperative dressing change. If dressing could keep dry and clean before discharge, patient wound received one dressing change. The dressing change caused by discharge was not recorded. All patients had antibiotics within 24 h, shower after 14 days and aspirin within 35 days postoperatively.

### Follow-up and wound evaluation

Each patient’s preoperative, intraoperative and postoperative information, including age, gender, body mass index (BMI), diagnosis, postoperative LOS, times of dressing changes and wound-related cost, were collected prospectively.

Wound-related cost was a sum cost of the suture, tissue adhesive, wound dressing, and other additional materials during the hospital stay.

At one-month follow-up, wound-related complications and evaluation scores were recorded. Wound-related complications included redness, dehiscence, subcutaneous hematoma, prolong wound drainage (> 5 days), surgical site infection (SSI) and re-suture caused by any reasons. Wound-related evaluation scores included patient scar assessment score (PSAS), Hollander wound evaluation score (HWES) and Vancouver scar score (VSS). Both two residents received professional training and stay satisfying consistency of wound evaluation in the pre-experiment. The mean scores evaluated by two resident were regarded as final scores. In addition, all patients would be asked to choose their preferred wound closure method.

PSAS [20]: the scoring system mainly refers to patient’s own feeling and evaluation of wound. Six represents normal skin and sixty represents worst imaginable scar (Table 1).

**Table 1:** Patient scar assessment score (PSAS)

^a^	1	2	3	4	5	6	7	8	9	10
Is the scar painful?										
Is the scar itching?										
^b^	1	2	3	4	5	6	7	8	9	10
Is the color of the scar different?										
Is the scar more stiff?										
Is the thickness of the scar different?										
Is the scar irregular?										
Total Score Patient Scar Score										

^a^ 0 means “no, no complains”,10 means “yes, more imaginable”

^b^ 0 means “no, as normal skin”,10 means “yes, very different”

HWES [21]: the scoring system includes 6 items, which are step-off of borders, contour irregularities, margin separation, edge inversion, excessive distortion and overall appearance. It was evaluated by two independent orthopedic residents unknown to the result of allocation. One point is for each item. The lower the score, the better the wound healing (Table 2).

**Table 2:** Hollander wound evaluation score (HWES)

Incision attribute	Score if absent	Score if present
Step-off borders	0	1
Contour Irregularities	0	1
Margin Separation	0	1
Edge inversion	0	1
Excessive Distortion	0	1
Overall appearance	0 (satisfactory)	1 (unsatisfactory)
Total Hollander score	0 (best)	6 (worse)

VSS [22]: the scoring system includes 4 items. They are vascularity, pliability, height and pigmentation. It was evaluated by two independent orthopedic residents unknown to the result of allocation. Lower scores represent a more normal appearance (Table 3).

**Table 3:** Vancouver scar score (VSS)

Score	Vascularity	Pliability	Height	Pigmentation
0	Normal	Normal	Flat	Normal
1	Pink	Supple	< 2 mm	Hypopigmentation
2	Red	Yielding	2-4 mm	Mixed
3	Purple	Firm	> 4 mm	Hyperpigmentation
4	–	Banding	–	–

### Statistical analysis

All statistical analyses were performed by SPSS version 22 (Inc., Chicago, IL, USA). Data was showed as median, mode and interquartile range (IQR) (skewed distribution) or mean ± standard deviation (SD) (normal distribution). Measurement data was analyzed by student’s tests or rank-sum test. Count data was analyzed by rank-sum test or Fisher exact test. A value of α = 0.05 suits all tests. The intraclass correlation coefficient (ICC) was used to assess the observers’ agreement: 0.81 to 1.00, nearly perfect reliability; 0.61 to 0.80, strong reliability; 0.41 to 0.60, moderate reliability; 0.21 to 0.40, fair reliability; and 0 to 0.20, poor reliability.

## Results

Thirty-three patients were enrolled in this study. Two patients failed to complete the scheduled follow-up and one patient had re-operation because of recurrent dislocation. Finally, 30 patients were analyzed, which included 23 patients with osteonecrosis of femoral head (ONFH), 5 patients with developmental dysplasia of hip (DDH) and 2 patients with ankylosing spondylitis (AS). Two AS patients had the non-steroidal anti-inflammatory drugs (NSAIDs) regularly for pain relief, and neither of them had the immunosuppressants or glucocorticoids between preoperative 1 month and postoperative 1 month. The basic information was shown in Table 4.

**Table 4:** The demographics of thirty patients

Basic information	Data
Age (Median, mode, IQR) (years)	30.5, 32, 11
Male:Female	17:13
BMI (Mean ± SD) (kg/m^2^)	22.94 ± 3.62
Postoperative LOS (Mean, mode, IQR) (day)	4, 4, 1

Three patients (four hips) had prolonged wound drainage and their basic information were shown in Table 5. The BMI of three patients was 28.50 ± 1.94 kg/m^2^ and that of remaining patients was 22.32 ± 3.21 kg/m^2^. The difference was significant (*p* = 0.003). There was no PJI during the follow-up period in this set.

**Table 5:** The information of three patients who had prolonged wound drainage

Sex	Age (years)	Diagnosis	BMI (kg/m^2^)	Hip	Wound closure	Treatment
Female	34	ONFH	27.13	Right	Standard	Re-suture and oral antibiotics
Male	31	DDH	27.65	Left	Standard	Re-suture and oral antibiotics
Male	37	ONFH	30.72	Right	Standard	Re-suture and oral antibiotics
–	–	–	–	Left	Tissue adhesive	Partial pressure bandage and oral antibiotics

**Fig. 2:**
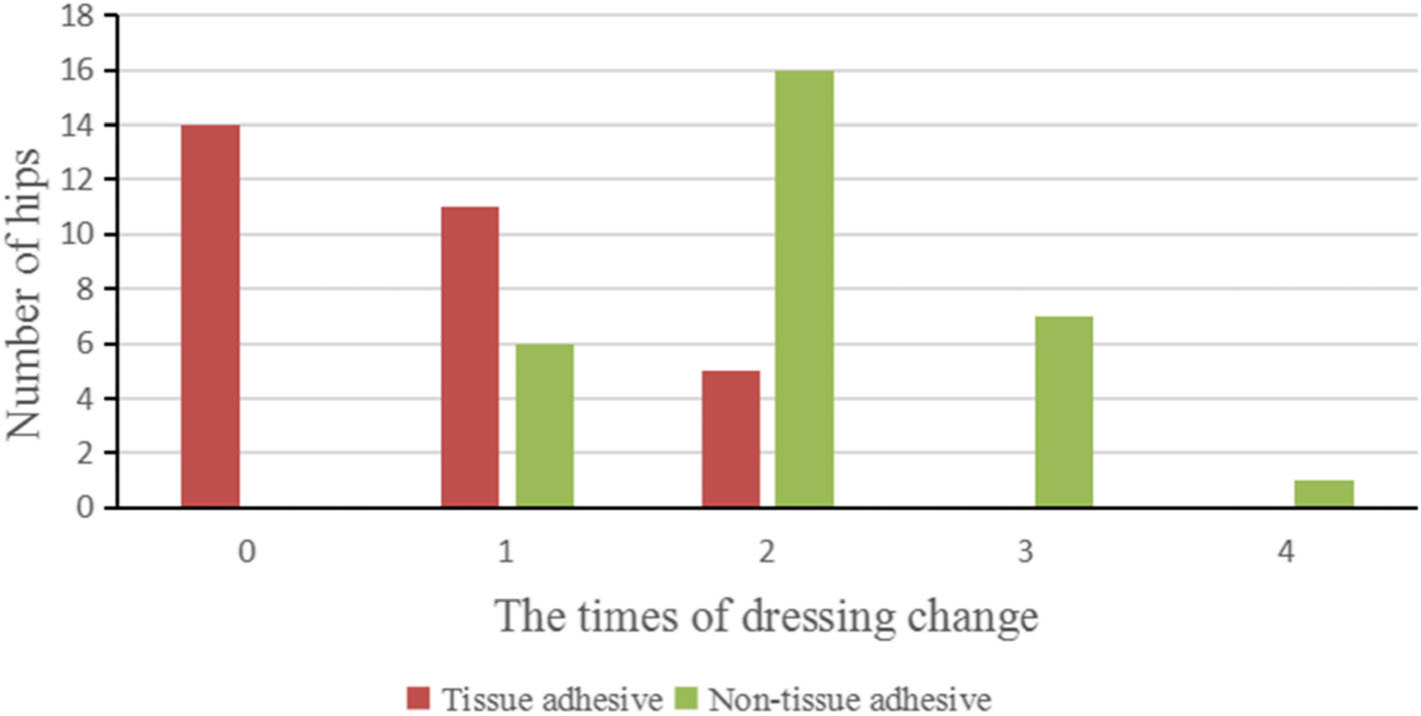
The times of dressing change between two methods in thirty patients

During the hospital stay, the times of dressing change in hips with tissue adhesive was significantly less than that in the other hips (*p* = 0.000) (Table 6, Fig. 2). Wound-related cost in hips with tissue adhesive was significantly higher than that in the other hips (*p* = 0.000).

**Table 6:** Dressing change, wound-related cost, complications and evaluation scores between two methods of wound closure in thirty patients

Data	Tissue adhesive	Standard wound closure	P
Dressing change (Median, mode, IQR)	0, 0, 1	2, 2, 1	0.000
Wound-related cost (Mean ± SD) (US dollar)	272.39 ± 10.12	221.83 ± 13.55	0.000
Wound-related complications	1/29	3/27	0.306
PSAS (Mean ± SD)	22.83 ± 9.48	30.57 ± 9.54	0.004
HWES (Median, mode, IQR)	0, 0,0	0, 0, 0	0.414
VSS (Mean ± SD)	5.13 ± 1.16	5.77 ± 1.16	0.057

**Fig. 3:**
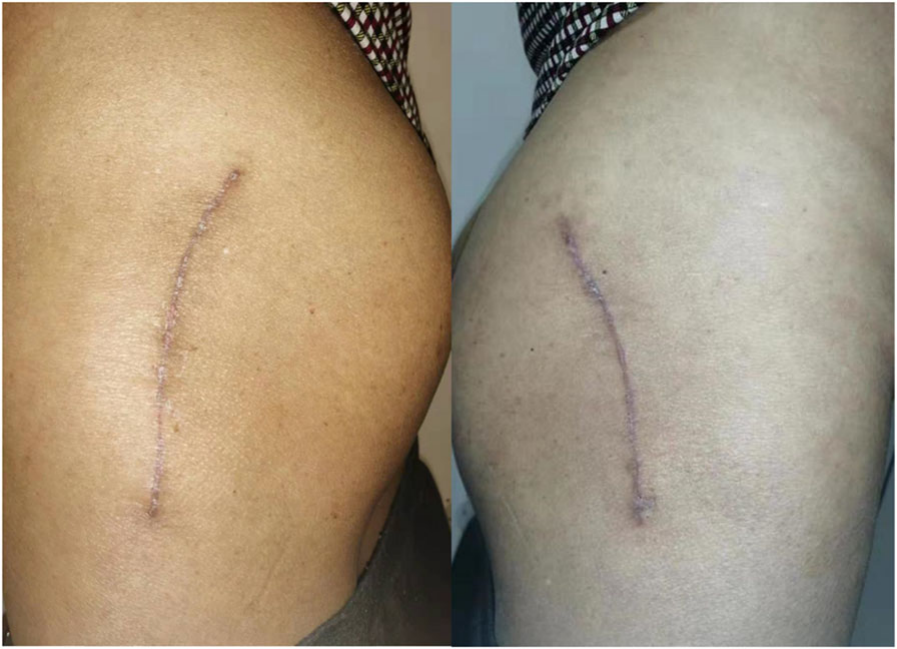
The preference distribution for wound closure in thirty patients

The postoperative one-month appearances of bilateral wound in one patient were shown in Fig. 3. From the view of patients, the PSAS in hips with tissue adhesive was significantly better than the other hips (*p* = 0.004). From the view of observers, there were no significant differences in the HWES or VSS between two methods (Table 6). These scores assessed by two observers had nearly perfect reliability with each other (ICC > 0.81) (Table 7).

**Table 7:** The inter-observers’ agreements between two observers

Evaluation system	PSAS, 95% CI	HWES, 95% CI	VSS, 95% CI
Inter-observer agreement	0.962 (0.931 to 0.965)	0.927 (0.892 to 0.964)	0.854 (0.755 to 0.910)

**Fig. 4:**
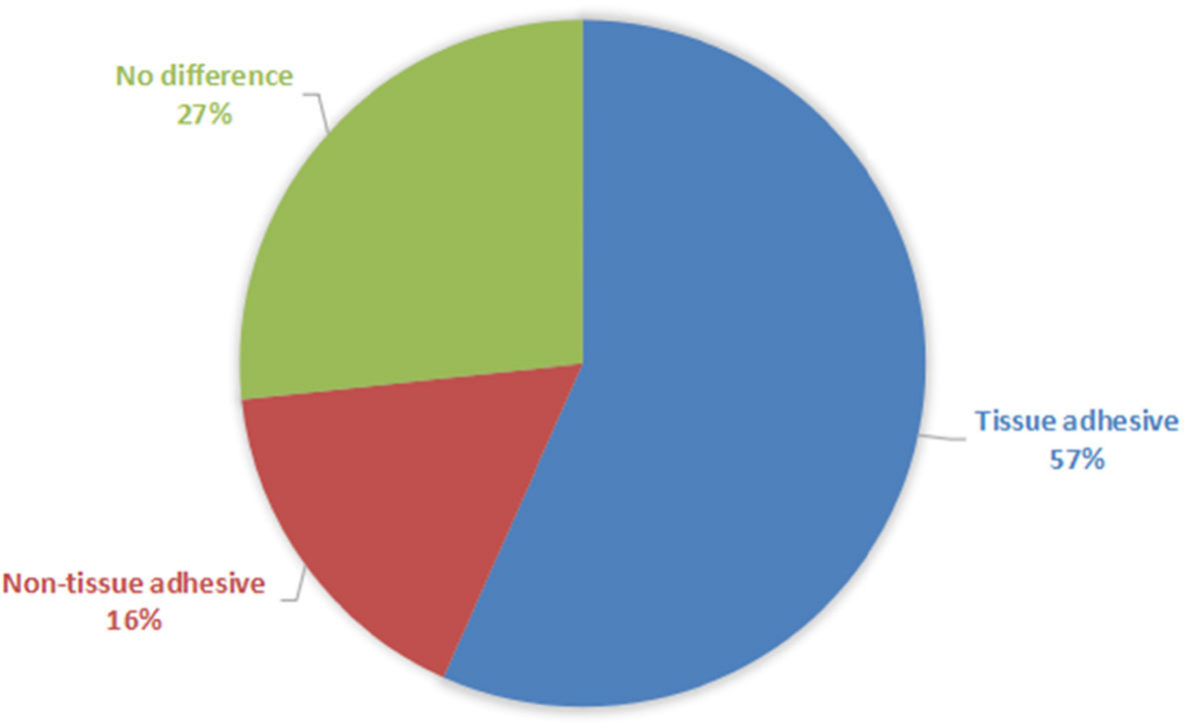
The appearances of bilateral wounds at postoperative 1 month (left: tissue adhesive; right: standard wound closure)

Based on the subjective feeling from patients, seventeen patients (57%) preferred the side with tissue adhesive, eight patients (27%) had no preference and only five patients (16%) preferred the other side (Fig. 4).

## Discussion

This prospective, randomized and self-controlled study found that the supplementary tissue adhesive to standard wound closure in hip replacement could reduce wound drainage and increase patient satisfaction, despite of increasing medical expense. So tissue adhesive combining with standard wound closure can be a reliable and effective choice for enhanced- recovery THA.

Since ERAS aims to improve wound healing, increase patients’ satisfaction and reduce hospitalization days, wound closure has become increasingly important.

In hip replacement, the most common wound closure methods are staples or sutures. Yet, no consensus has been reached in the literature as to which closure method is better [23]. From the perspective of time usage, skin staples requires less time than suture [24]. From the perspective of wound complications, one meta-analysis including several high-quality studies found that, when compared with sutures, skin staples had significantly higher risk of developing wound infection. Furthermore, this risk was especially greater in hip surgery [25]. From the perspective of wound healing, running subcuticular suture proved to ensure more physiologic robust blood flow than vertical suture and skin staples, which was one important factor in determining wound healing [26].

In our institute, the patients who underwent THA were relatively young, and had the higher expectation on wound appearance and enhanced recovery.

Many studies had conformed the role of subcuticular suture in improving incision appearance after joint surgery [27–29]. For cosmetic appearance, we usually adopted subcuticular suture, rather than skin interrupted suture and staple. However, this standard wound closure failed to solve the elementary problem of wound drainage.

Prolonged wound drainage could be detrimental to clinical outcomes [11, 30]. Therefore, wound management urgently needs new techniques and materials.

Tissue adhesive is probably a good choice. The tissue adhesive in this study is composed by N-butyl-2-cyanoacrylate and early application in humans can be traced back to 1980s [31]. It would form a protective film on the surface of wound in 30 s and then provide enough adhesive strength. Meanwhile, it has certain bacteriostatic action. Therefore, these characteristics and advantages contribute to its extensive clinical application in surgery.

In this study, the times of dressing change in thirty hips with tissue adhesive decreased by 50% when compared with the other thirty hips. The patient-reported evaluation scores of wounds with tissue adhesive were better than those without tissue adhesive. In the group of standard wound closure, the patients who had bilateral posterolateral incisions needed to lie on their side for 3–5 min to finish disinfection and dressing change, which would aggravate the pain of the other incision. But in the group of tissue adhesive, less dressing change could reduce surgeons’ workload and inconvenience to patients. This might explain why number of patients preferred tissue adhesive were three times as many as standard wound closure.

Some surgeons have also reported their result of using tissue adhesive in arthroplasty. Gromov and El-Gazzar proved its role in reducing wound drainage when tissue adhesive worked as the supplement to staples [8, 11]. However, another two randomized controlled trials showed no significant differences in cosmetic appearance of scars, incidence of complications, and patient satisfaction between tissue adhesive and standard wound closure [10–12]. These authors just analyzed the clinical outcomes, without taking dressing change and medical expenses into consideration. According to the above studies and our experience, tissue adhesive should be the adjunct, not substitution, to standard wound closure in THA.

There is no denying that some patients were allergic to tissue adhesive, though no allergy was found in this study [32, 33]. Dunnett performed 912 knee replacement and discovered allergy in 1.7% patients [34]. It usually manifested as erythematous pruritic papular rash surrounding incision site within postoperative 3 weeks.

Although tissue adhesive could reduce wound drainage and increase patients’ satisfaction, we should be aware of possible risks of wound complications. Wood et al. reported that the time taken for wounds to stop oozing following hip replacement was significantly related to BMI [35]. Zhang et al. also found that patients who were obese (BMI > 30 kg/m^2^) had higher odds of wound complications in both non-urgent and urgent orthopedic surgery [36]. This study also showed that obesity might be a risk factor of wound complications.

This study has several strengths. Firstly, randomized controlled trial could reduce selection bias, information bias and confounding bias, which was unavoidable in case-control study. Secondly, self-controlled study could balance the bias between comorbidities and physical conditions among individuals. Thirdly, patients and residents evaluated wound respectively. Fourthly, the study took economic factors into account.

This study is not without limitations. Firstly, since this study aimed to investigate wound closure on enhanced-recovery THA, we only followed up patients in postoperative 1 month. Small sample size and short follow-up period were not sufficient for detailed and comprehensive analysis. Secondly, patients who can bear bilateral hip replacements simultaneously usually have good physical conditions. The result remains unknown in application of tissue adhesive for less healthier populations. Thirdly, we didn’t conduct mechanical tests to measure the adhesive strength of tissue adhesive, which may influence the persuasiveness of this study. Fourthly, wound closure time weren’t recorded, which made it impossible to compare the influence on operating time. Fifthly, medical consumptive material pricing system in our institutes might be different from others, so the cost-performance of tissue adhesive would vary among institutes.

## Conclusions

Tissue adhesive could significantly reduce wound drainage and increase patients’ satisfaction, which can be an ideal adjunct to standard wound closure in enhanced-recovery THA.

## Data Availability

The datasets used and/or analysed during the current study are available from the corresponding author on reasonable request.

## Abbreviations

THA: Total hip arthroplasty
BMI: Body mass index
LOS: Length of stay
ERAS: Enhanced recovery after surgery
PJI: Periprosthetic joint infection
TKA: Total knee arthroplasty
TXA: Tranexamic acid
SSI: Surgical site infection
PSAS: Patient scar assessment score
HWES: Hollander wound evaluation score
VSS: Vancouver scar score
IQR: Interquartile range
SD: Standard deviation
ICC: Intraclass correlation coefficient
ONFH: Osteonecrosis of femoral head
DDH: Developmental dysplasia of hip
AS: Ankylosing spondylitis
